# Transboundary Animal Diseases and Human Migration: A One Health Perspective on the Balkan Route

**DOI:** 10.1155/tbed/5272522

**Published:** 2026-02-13

**Authors:** Eleonora Uber, Giorgia Angeloni, Angelo Peli, Alessandra Mistral De Pascali, Ludovica Ingletto, Alessandra Scagliarini

**Affiliations:** ^1^ DVM Graduate, Department of Veterinary Medicine, University di Bologna, Ozzano dell’Emilia, 40064, Italy, unibo.it; ^2^ Istituto Zooprofilattico Sperimentale delle Venezie, Viale dell’Università, 10, Legnaro (PD), 35020, Italy, izsvenezie.it; ^3^ Department for Life Quality Studies, University of Bologna, Rimini, 47921, Italy, unibo.it; ^4^ Department of Medical and Surgical Sciences (DIMEC), University of Bologna, Bologna, 40138, Italy, unibo.it

## Abstract

Transboundary animal diseases (TADs), including zoonoses, can be introduced in non‐endemic areas through animal trade and uncontrolled movements during times of conflict and migration. Sheep and goat pox (SGPX) and peste des petits ruminants (PPRs) were, respectively, reported in Europe in 2018 and 2010. EFSA emphasised the risk of spread into Europe through informal trade and unmonitored migration routes that may involve the informal transport of small ruminants. Using a One Health conceptual framework, which considers the interconnectedness of human, animal, and environmental health, this study analysed the epidemic trends of the two selected diseases during 2014–2025, with particular focus on the role of human migrations in influencing their spread along major migratory routes in the Balkan Peninsula. The conceptualisation of animals in migration settings was investigated through the scrutiny of policies and semi‐structured interviews to experts and people who travelled the route. The presence of small ruminants, companion and pest animals was confirmed in formal and informal camps. The results underscore the relevance of animals in the migration contexts of the Balkan routes, as well as the risks associated with their presence in settlements in the absence of an adequate management. Our findings seem to suggest that human migration along the Balkan routes is not directly responsible for the spread of TADs, as there is no evidence of significant animal movements accompanying migrants. However, our results show a possible role of informal trade networks following human migration paths along the Balkan route, while a possible role of informal trade networks, following human migration paths along the Balkan route, deserves to be further investigated.

## 1. Introduction

The Balkan route represents a critical corridor for displaced populations seeking entry into Europe and it is characterised by a complex mix of geopolitical, economic and socio‐cultural dynamics [1, 2]. The migration route across the Balkan Peninsula represents one of the major land pathways to Europe. Migrants travelling across this route often experience prolonged transit, spending extended periods in both formal and informal camps [3–6]. Human migration has been associated to the spread of transboundary animal diseases (TADs) with high socio‐economic impact; in particular, small ruminants can be transported across borders during times of conflict and migration [7, 8]. This study focuses on small ruminant TADs peste des petits ruminants (PPRs) and sheep and goat pox (SGPX). PPRs is a highly infectious viral disease that affects primarily small ruminants with high morbidity and mortality [9] and devastating impact in affected areas, compromising agro‐pastoral communities [10–13]. South‐Eastern European countries have been affected by PPR outbreaks, with the first cases in Europe reported in Bulgaria in 2018. Literature highlights the role of markets and communal grazing areas in the increase transmission risks, while trades and uncontrolled animal movement have been recognised as the main pathway of introduction into non‐endemic areas [14–17]. However, the potential role of human migration in facilitating the transboundary spread of PPR into Europe through the Western Balkans still needs to be assessed.

SGPX affects sheep and goats, causing skin lesions, fever and potentially fatal infections [18–20]. Outbreaks result in production losses, such as reduced milk yield, wool quality and higher mortality, as well as indirect losses due to trade restrictions [21–23]. The disease impacts global trade, hinders national economies, particularly in developing countries. Similar to PPR, it poses a major threat to agro‐pastoral communities that depend on small ruminants for income and food security [18, 19]. The virus spreads through direct and indirect contact due to high environmental resistance. Indirect transmission can occur via contaminated surfaces, water, feed and wool, contributing to the virus’s potential for endemicity [19, 20]. Uncontrolled animal movement constitutes a major pathway of transmission with illegal trade, pastoralism and migration associated with SGPX outbreaks. These risks are heightened by human migrations, which may involve the informal transport of animals. For instance, outbreaks reported in Greece and Bulgaria in 2013 and 2014 were associated with the movement of Syrian refugees fleeing the 2012 civil war and crossing into Europe via Turkey [24].

SGPX is endemic in several countries of the African continent, Central Asia and the Indian subcontinent, with cases also reported in regions neighbouring the Western Balkans, such as the Middle East [22, 25]. Both Greece and Bulgaria have experienced SGP outbreaks in the 2010s, underscoring the risk of spread into the European Union.

The aim of this study was to investigate the role of animals in human migrations through the Balkans, their presence in formal and informal settlements and the risks of introduction of the three selected TADs associated with uncontrolled movements. With this aim, a disease event analysis was performed to map disease occurrence and identify any possible overlap between outbreaks and migratory corridors along the Balkan route. Discourse analysis on migration health policies in place was carried out to investigate the institutional narratives regarding animals in migration. Finally, semi‐structured interviews were performed to understand how animals are perceived by migrants who travelled the route, as well as humanitarian and health professionals operating in camps.

## 2. Materials and Methods

### 2.1. Study Area

**Figure 1:**
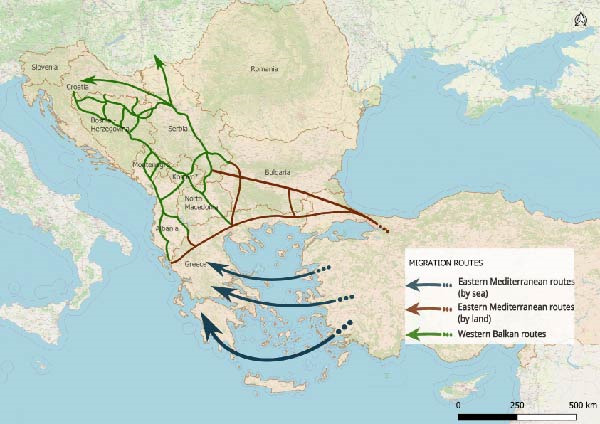
Main migration routes in Western Balkans and Eastern Mediterranean region. This map illustrates the principal migration routes of the Western Balkan and Eastern Mediterranean corridors, based on data from the International Organization for Migration (IOM) [27]. The map was created in QGIS v.3.34 (https://qgis.org/) in accordance with United Nations Security Council Resolution 1244 [1999]).

The study focused on the countries crossed by the Balkan route, namely, Albania, Bosnia and Herzegovina, Kosovo ^∗^, Montenegro, North Macedonia and Serbia [26]. Greece and Bulgaria, despite formally belonging to the Eastern Mediterranean route, are commonly considered part of the Balkan route, and for this reason included as depicted in Figure 1.

Given the dynamic, fragmented and inherently informal nature of the Balkan routes [2, 6], it is highly challenging to map them comprehensively, thus, only major known pathways are depicted in Figure 1.

### 2.2. Disease Events Analysis

Migration routes were reconstructed from non‐GIS maps provided by the International Organization for Migration (IOM). These maps were georeferenced in QGIS v.3.34 (https://qgis.org/; EPSG:3857), and approximate route vectors were manually digitised. Outbreak data (01/01/2014–31/072025) on PPR and SGPX in the study area were downloaded from the World Animal Health Information System (WAHIS) database (https://wahis.woah.org/#/event-management, retrieved via the public API last accessed on 01/11/2025). Outbreak data were imported into QGIS and grouped into six‐time intervals (2014–2015, 2016–2017, 2018–2019, 2020–2021, 2022−2023 and 2024–2025). Data were visualised in conjunction with migration routes to assess potential spatial overlap between disease outbreaks and migratory corridors, with the aim of producing exploratory maps for visual analysis.

### 2.3. Qualitative Data Collection on the Presence, Management and Role of Animals in Displacement Contexts

#### 2.3.1. Policy Analysis

A discourse analysis of policies governing health in migration was performed to understand how the presence of animals (and their health) is conceptualised in institutional narratives. The research included guidelines and standards of international organisations, supranational bodies, non‐governmental organisations (NGOs) reports and specific policies of Balkan countries, to have a multi‐level understanding. All documents included in the analysis were published in English, as the most relevant materials on migration and animal health along the Western Balkan route—those issued in local languages—were not publicly accessible.

Policies scrutiny was conducted using the Bacchi’s ‘What is the problem represented to be’ (WPR) discourse analysis tool [28]. This method focuses on how policy constructs problems, rather than simply responding to them, by interrogating the implicit assumptions and consequences embedded in policy discourse. This tool relies on six different questions to guide the analysis. For the study, three questions were applied to critically analyse the policies: (1) What is the ‘problem’ represented to be in the most relevant policies. (2) What presuppositions or assumptions underlie this representation of the ‘problem’. (3) What effects are produced by this representation of the ‘problem’. These three questions were used to understand how animals are conceived and how this reflects on their management in the context of Balkan route migration. The first two questions help to uncover the underlying logic and assumptions within policy texts, while the third considers the material and discursive effects of these representations.

#### 2.3.2. Semi‐Structured Interviews With Humanitarian and Health Professionals

Semi‐structured interviews were carried out with humanitarian, health and NGOs workers, researchers and camp coordinators involved in the Western Balkan route to assess the presence of animals in camps, how animals are perceived by humanitarian professionals and to what extent the presence of animals can be associated with the health and welfare of migrants and refugees. A purposive and snowball sampling strategy [29] was used to recruit humanitarian and NGOs workers, researchers and camp coordinators, selected for their experience along the Western Balkan route. Interviews focused on the presence of animals in displacement settings, their perceptions and management practices (Supporting Information 1). The interviews were audio‐recorded with the participants’ consent, and subsequently transcribed verbatim for analysis. Interviews were analysed thematically using a hybrid coding approach. A set of deductive codes was developed a priori based on the socio‐ecological system framework (SESF). This framework was used as a tool to analyse how socio‐political, environmental and biological factors, interacting at five different hierarchical levels (individual, interpersonal, community, organisational and policy level), may act as interdependent determinants to facilitate the diseases spread in migration settings [30, 31]. In particular, the SESF was adapted to the context of migrant camps [32] and integrated with the specific risk factors of the investigated diseases, as shown in Table 1.

**Table 1:** Variables and respective items in the modified SESF for infectious diseases, including transboundary animal diseases, within migrant camps on the Balkan Route (adapted and integrated from Peli [32]).

Variable name	Items	Levels of the SESF
Camp and camp‐like settings	Population density; institutional settlement; informal settlement; ecosystem disruption/population; rearrangement	Interpersonal; community; organisational
Animals presence	Uncontrolled movement of animals; animal health status and management; common grazing; husbandry method; wildlife interactions; presence of vectors	Policy; organisational; community; individual
Housing standards	Brick; mud; tent; shack; container	Policy; organisational; interpersonal
Water resources	Water supply system; waste‐water management; waster wells, streams, dams; sharing with livestock and animals	Organisational; interpersonal; individual
Politics	Exclusive norms; inequalitarian policy system; border control loosening; hierarchy issues	Policy; organisational; community
Pathogen related	Genomic variability; species jumping; resistance; vector transmission	Individual; interpersonal; community
Culture and behaviour	Reliance on livestock; transport of animal origin goods; consumption of raw meat/milk/animal products; harvesting close to the camp; trade and market with local communities	Policy; organisational; community; interpersonal; individual

In parallel, inductive codes were generated directly from the data to capture emerging themes and unexpected patterns. Coding was carried out manually and iteratively refined during multiple readings of the transcripts (Supporting Information 2). One researcher led the coding process, with periodic discussions with a second reviewer to enhance consistency and reduce bias. Informed consent was secured from all participants prior to data collection. All the participants were provided with detailed information regarding the study’s aims, the voluntary nature of their participation and their right to withdraw at any time. Confidentiality was ensured through pseudonymisation of interview transcripts and secure data storage procedures.

#### 2.3.3. Informal Conversations With Migrants

To explore the roles of animals during migrants’ journeys, informal conversations and a multi‐lingual open‐ended questionnaire were conducted. This phase sought to unpack the underlying mechanisms that shape the role of animals in displacement settings. In particular, the conversations were aimed to assess the extent to which animals accompany people during migration and their presence in the displacement settings along the Balkan route. Beyond their potential role in disease transmission, interviews were aimed at assessing how animals contribute to food security, economic resilience and psychological well‐being in these contexts. Interviews were conducted in Trieste (Italy), a key junction of the Balkan route. Conversations with migrants (regardless of their legal status) were facilitated by cultural mediators to explain the research aims, assess interest in participation and address any questions. A multi‐lingual online questionnaire was then offered to those who voluntarily expressed interest (Supporting Information 3). The questionnaire focused on animal presence and role during migration, without collecting sensitive personal data. Ethical safeguards included voluntary participation, anonymity and the right to withdraw. Given the vulnerabilities of this population, the research design prioritised trust‐building and avoided recording or collecting identifiable data.

### 2.4. Ethical Considerations

Ethical approval for this study was obtained from the Bioethics Committee of the University of Bologna (Protocol Number 0070282 on 12/03/2024).

## 3. Results

### 3.1. Disease Events Analysis

The spatio‐temporal evolution of PPR and SGPX in South‐Eastern Europe is jointly illustrated in Figure 2. The maps show the cumulative distribution of outbreaks aggregated by biennium, from 1 January, 2014 to 31 July, 2025. Outbreaks or cases reported in previous periods are retained in subsequent maps to highlight the progressive expansion and geographical persistence of these diseases overtime. For PPR, no events were recorded in Europe until June 2018, when the first outbreaks were reported in the Yambol and Burgas regions of Bulgaria. After a period of apparent absence, further outbreaks re‐emerged in July 2024 in Greece and Romania. In 2025, additional reports were collected in Romania as well as, first occurrences in Hungary and Albania. SGPX outbreaks were first detected in Greece in 2007 (Lesvos Island), with recurrent episodes in mainland Greece and Bulgaria until 2017. After a 5‐year hiatus, new cases emerged in Bulgaria and Greece in 2023, followed by widespread notifications in 2024–2025, including the first official reports in Romania.

Figure 2Epidemic trends of PPR and SGPX, in South‐Eastern Europe, 2014–2025. The maps show the cumulative spatial distribution of outbreaks (PPR and SGPX): (A) 2014–2015; (B) 2016–2017; (C) 2018–2019; (D) 2020–2021; (E) 2022–2023; (F) 2024–2025. Outbreaks reported in previous biennia are retained in subsequent maps to visualise progressive geographic expansion (outbreaks happened prior than 2014 were not included in the map). Circles represent disease occurrence, scaled to the number of reported outbreaks per subregion. Arrows indicate the main migratory routes, as described in Figure 1. PPR and SGPX outbreak data were retrieved from the World Animal Health Information System (WAHIS) of the World Organization for Animal Health (WOAH). The maps were created in QGIS v.3.34 (https://qgis.org/). Data sources: WOAH‐WAHIS [33]. Last accessed on 01/11/2025.

**Figure:**
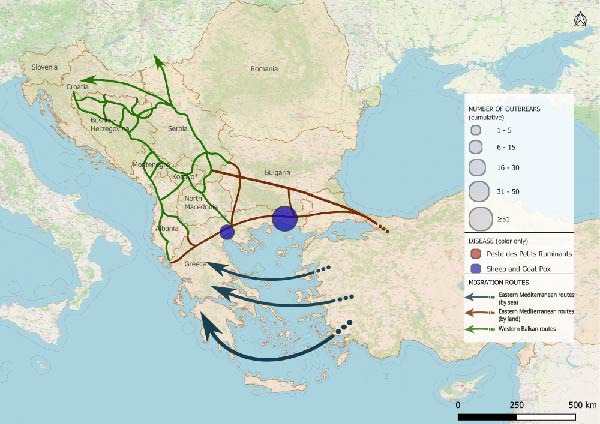
(A)

**Figure:**
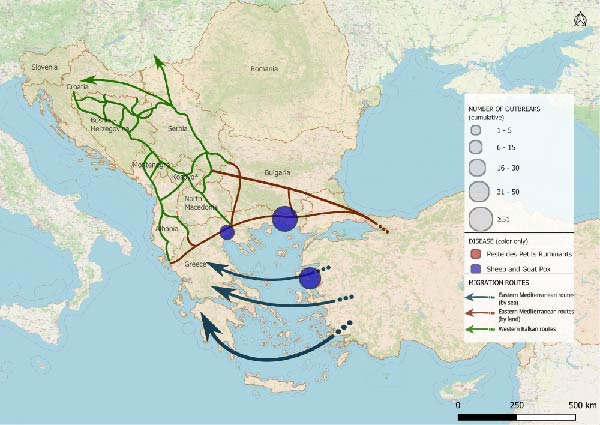
(B)

**Figure:**
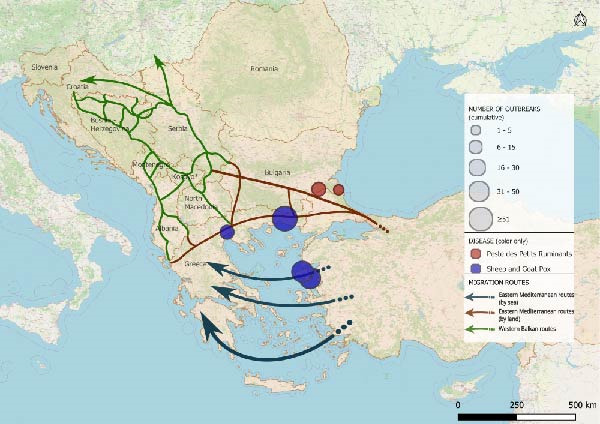
(C)

**Figure:**
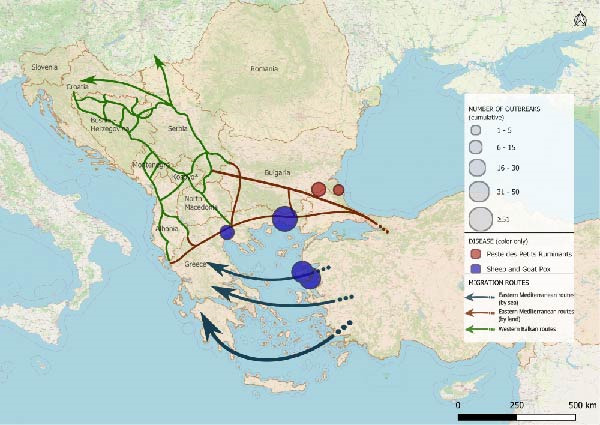
(D)

**Figure:**
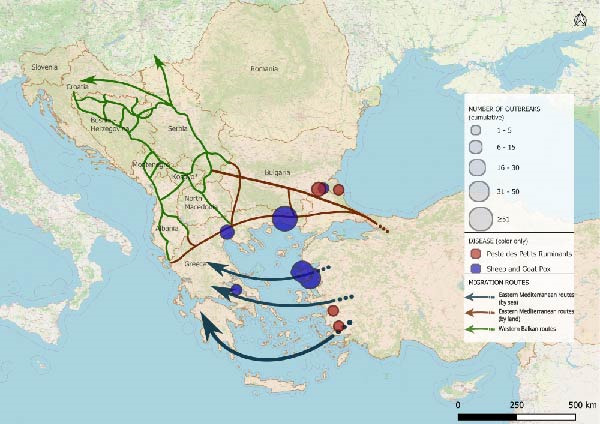
(E)

**Figure:**
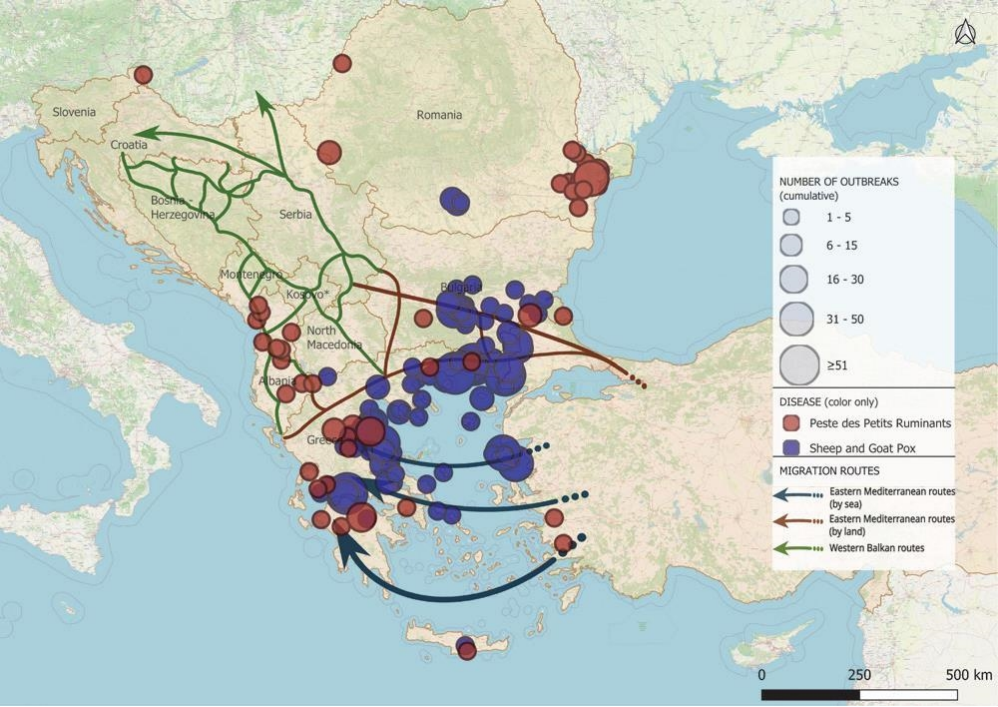
(F)

Biennial non‐cumulative maps can be found in Supporting Information 4. Additionally, annual outbreak count per Country can be found in Supporting Information 5.

### 3.2. Policy Analysis

The healthcare landscape on the Balkan route is the result of a complex multi‐stakeholders’ system with intricate relationships and hierarchies. These linkages yield a complex web, which can vary on a ‘case by case’ basis, depending on various factors including the country under consideration, the national law, the international organisation and NGOs involved. Despite the diversity of actors engaged in managing migrants’ health, a common thread, across these systems, is the consistent neglect of animal presence and health, or their consideration solely in terms of the potential disease risk posed to humans. Table 2 reports a list of relevant policies and stakeholders and the main findings on the role of animals in migrants’ health and welfare.

**Table 2:** Summary of available policies and stakeholders governing health in the context of Balkan migration.

Policy/document/stakeholder	Main findings on animal health
WHO Global Action Plan on Refugee and Migrant Health (2019–2023, extended to 2030)	No reference to animal health, despite mentioning a holistic approach [34]
WHO Strategy and Action Plan for Refugee and Migrant Health in the European Region (2023–2030)	Acknowledges social, economic and environmental health determinants, but does not mention animals [35]
UNHCR Global Strategy for Public Health (2021–2025)	Animals are only mentioned as disease vectors without mentioning their health management [36]
UNHCR Emergency Handbook	Outlines standards for health and WASH in camps, but no reference to animal health or animal management [37]
New Pact on Migration and Asylum (European Commission, 2020)	Focuses on human health checks and integration. No mention of animal health [38]
WHO—Bosnia and Herzegovina: assessing health systems capacity to manage large influx of refugees and migrants (2020)	All health services provided by local health workers. No mention of animal health, veterinarians not included among health workers [39]
WHO—Greece: assessing health systems capacity to manage large influx of refugees and migrants in an evolving context (2020)	Health services provided by national system and NGOs. No reference to animal health, veterinarians not included among health workers [40]
NGOs (e.g., MSF, Médecins du Monde)	Provide a wide range of human health services. No reference to animal health or animal presence [41–44]

### 3.3. Interviews With Humanitarian and Health Professionals

Interviews were conducted between March and November, 2024. Overall, 18 professionals agreed to participate in the study. Their background and roles in the Balkan migration route are summarised in Table 3.

**Table 3:** Pseudonymised professional profiles of interviewed experts, reporting background, role, geographical areas of expertise and experience in formal and informal camps.

Pseudonym author	Professional background	Role in the Balkan context	Areas of expertise	Experience in formal camps	Experience in informal camps
A1	Political science and international relations former student	NGOs operator	Bosnia 2022 Bulgaria 2023	No No	Yes Yes
A2	Master in migration studies	Field coordinator	Greece 2019 Greece 2022 Greece 2023–ongoing	Yes Yes Yes	No No No
A3	BS psychological science and techniques	NGOs operator	Serbia 2023	No	Yes
A4	BA community psychology	NGOs operator	Greece 2022 Serbia 2023	Yes No	No Yes
A5	Business management and retail	CEO of UK charity	Greece 2016–ongoing	Yes	No
A6	Psychologist and psychotherapist	Field coordinator	Greece 2021–2023	Yes	No
A7	Educator for work integration of vulnerable categories	NGOs coordinator	Serbia ^∗^, Bosnia ^∗^, Bulgaria ^∗^2018–ongoing	Yes	No
A8	Relief worker	NGO field coordinator	Bosnia ^∗^2016 Serbia 2017 Bosnia 2019–ongoing	Yes Yes Yes	Yes Yes Yes
A9	No background	Humanitarian volunteer	Greece 2019 Bosnia 2020	Yes No	No Yes
A10	PhD candidate in gender studies	Teacher	Greece, 2019–2023	Yes	Yes
A11	—	Humanitarian volunteer	2019 Serbia 2021 Bosnia 2023 Greece	Yes Yes Yes	No No No
A12	Journalist	Journalist	Bosnia ^∗^, Serbia ^∗^, Greece ^∗^, Ukraine	Yes	No
A13	No background	NGO field coordinator	Ukraine	—	—
A14	Nurse	NGO nurse	Greece, 2019	Yes	No
A15	Refugee history scholar	—	—	—	—
A16	Global health and displacement scholar	Researcher	Greece, 2018	Yes	No
A17	Refugee health and global health scholar	Researcher	Bosnia, 2022	Yes	No
A18	Red cross veterinarian	—	—	—	—

*Note:* ‘–’, no experience in the column reported. In columns ‘formal camps’ and ‘informal camps’, explains the context of the described experience.

^∗^Expert that dynamically worked in the mentioned countries in the mentioned years, not only in a specific camp.

**Figure 3:**
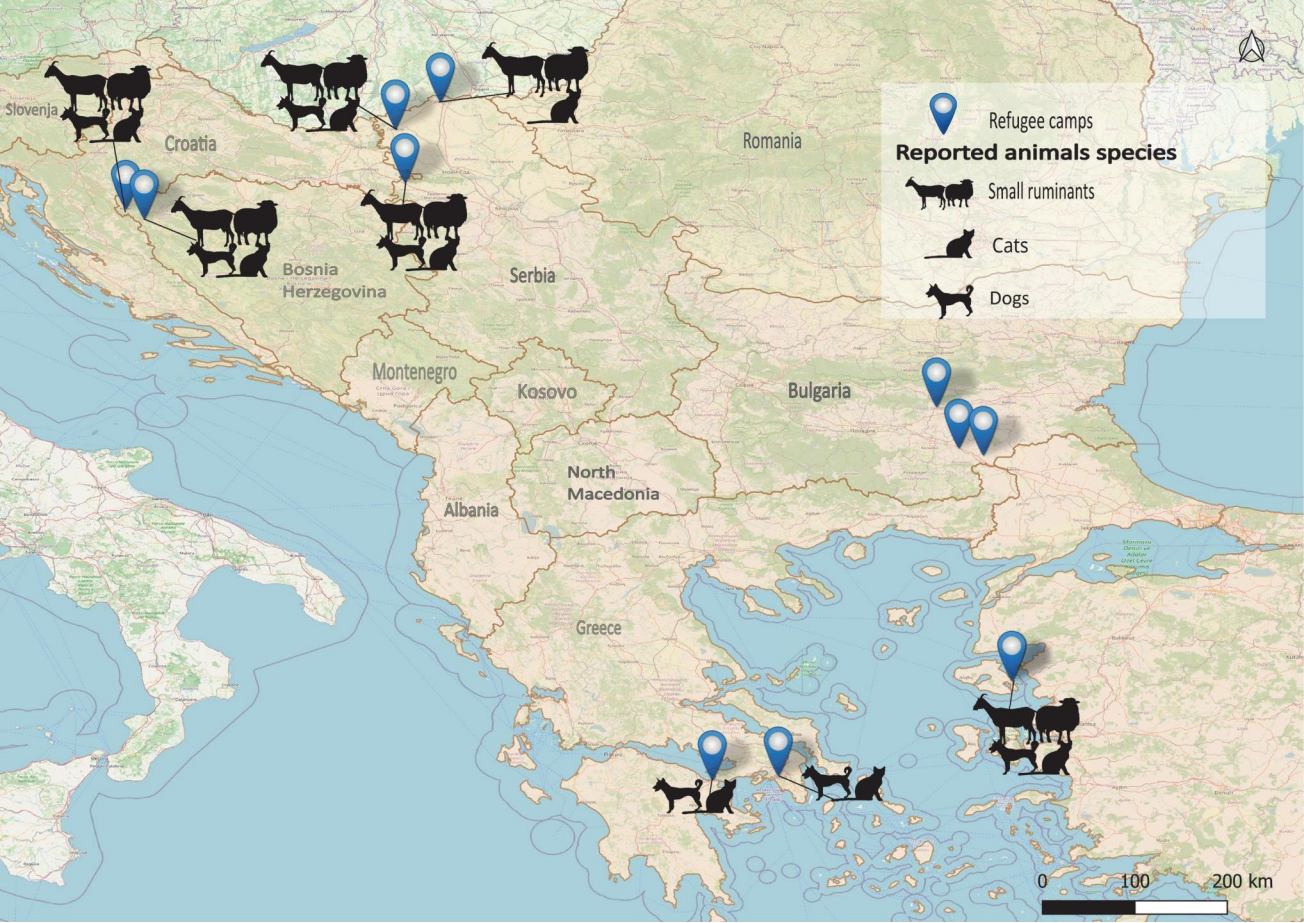
Camps and animal species reported along the Balkan migration route. The map shows the approximate locations of both formal and informal camps based on the respondents’ experiences, along with the animal species they reported. The locations were directly provided by the respondents themselves. The map was created in QGIS v.3.34 (https://qgis.org/).

The map provides the approximate location of camps, both formal and informal, where the respondent worked (Figure 3). The localisation on the map was provided by respondents themselves.

Interviewees confirmed a widespread presence of animals in both formal and informal camps across Greece, Serbia, Bosnia and Bulgaria. Two distinct categories of animals were identified through the interviews: ‘pets’ (cats and dogs) and small ruminants (goats and sheep). These animals were observed both in informal and formal camps. In informal camps, the absence of oversight allows residents to independently decide whether to coexist with these animals. Differently, in formal camps, animals are technically not allowed by regulations. However, their presence is tolerated if it remains ‘under control’ in terms of numbers and safety (A8). Interviewees consistently reported that both animals originated locally rather than brought from countries of origin, as confirmed by migrants’ questionnaire, where few of the respondents recalled meeting someone who brought animals with themselves on the migration. Several experts (A1, A2, A3, A4, A5, A6, A7, A8 and A9) explained that the dogs and cats are mostly stray animals that get closer to the camps due to presence of food. Only two respondents (A8 and A5) reported migrants travelling with pets from their country of origin, primarily dogs, cats and in one case, a rabbit. None of the respondents recalled people migrating with small ruminants. Interviewers A1, A3 and A4 reported that the procurement of sheep and goats often depended on the presence of ‘smugglers’ in the area. These individuals, being familiar with the region and its resources, would handle animal acquisition, although none of the respondents could provide further details on the exact origins of the animals. The local Islamic community can play a critical role in animal supply, as illustrated in the Bosnian context. In fact, during religious celebrations, small ruminants are sometimes donated to formal camps, where residents independently slaughter them the following day (A8). Several interviewees described the occurrence of ritual slaughter during Islamic celebrations such as Eid al‐Adha. The cultural value of animals is also prominent in informal camps in Bosnia and Serbia, where ritual animal slaughtering is practiced, particularly during religious celebrations such as Eid al‐Adha (A1, A3, A4 and A7), though not exclusively, as noted by A4.

In formal camps, such practices were reported only by two respondents (A5 and A8), but did not exclude it could have happened outside the camp. Additionally, food items of animal origin, such as dried meats and cheeses, were sometimes brought by (rare) migrants travelling by plane or (mostly) shipped by relatives (A8). These goods, usually unlabelled and non‐regulated, represented a connection to cultural identity and home‐country practices. As a confirmation, none of the migrant respondents provided information about the possibility of bringing animal products from origin country along the route.

Interviewees emphasised that the presence of animals was often related to the duration of their stay (A15, A16 and A17). People in transit rarely adopted or cared for animals, while those who remained longer developed bonds or allowed animals to live nearby. A18 pointed out that, despite the transient nature of the route, the fact that is continuative, makes it sort of permanent. No veterinarians or animal health services were reported along the route, and none of the experts recalled formal veterinary interventions. Confirmed by the migrants that met through their journey only physicians. Ritual slaughters and animal cohabitation occurred without inspections or health controls (A7, A1, A3 and A4). One veterinarian interviewed (A18) described this absence as a form of ‘wilful neglect’, a deliberate institutional decision to avoid formalising animal presence, especially in informal camps, thereby avoiding additional responsibilities concerning the management of informal camps. Respondents noted that while zoonotic disease risks might exist, they were not considered a priority, either by institutions or by migrants themselves.

In contrast, most of the respondents identified overcrowding, inadequate sanitation, precarious food provision and insufficient healthcare as the most urgent concerns across both formal and informal camps (A7, A9, A10, A11, A12, A14, A16 and A17). While informal settlements lacked basic facilities such as running water or toilets, even formal camps struggled with waste management, pests and food insecurity (which was mostly NGOs provided; A1, A3, A4, A7, A8, A10, A11 and A12). Healthcare provision, when available, was described as fragmented and largely dependent on NGOs (A2, A5, A7, A9, A12 and A14), whose presence was often under‐resourced, while access to public health services was inconsistent and sometimes marked by hostility from medical staff (A2, A4 and A10). These conditions were perceived as immediate threats to well‐being and, thus, regarded as higher priorities than animal health.

### 3.4. Informal Conversations With Migrants

Overall, a total of five testimonies from migrants who have travelled the route were collected. Table 4 shows nationalities and details of their route when possible.

**Table 4:** Pseudonymised information about respondent.

Pseudonym	Country of origin	Year left the country	Countries crossed
B1	Afghanistan	2015	Pakistan, Iran, Turkey, Greece, Italy
B2	Syria	2016	No answer
B3	Morocco	2017	Turkey, Greece, Albania, Macedonia, Montenegro, Romania, Serbia, Bosnia, Croatia, Hungary, Slovakia, Slovenia, Italy
B4	Afghanistan	No answer	No answer
B5	Afghanistan	No answer	No answer

*Note:* It summaries respondents’ country of origin, year of arrival in Europe and countries reportedly crossed along their migration journey. Where the respondent did not provide an answer to a specific question, this is indicated as ‘No answer’.

The limited number of migrant interviews reflects the structural difficulty of conducting qualitative research with undocumented or transit migrants, who often experience mistrust, confidentiality concerns and fear of legal repercussions [2, 45–48]. Despite the small number, these interviews were retained because they provided essential first‐hand insights into the presence and role of animals during migration. Importantly, their accounts were not used in isolation, but systematically triangulated with those of aid workers and other respondents; this process cross‐checks themes across different data sources, strengthening credibility and completeness of qualitative findings [49–51]. None recalled meeting someone who brought animals with themselves on the migration. All the respondents mentioned the presence of ritual slaughter, in the occurrence of Eid al‐Adha, Eid al‐Fitr and birthdays. B4 and B5 specified that these slaughters occurred in Serbia. In a camp on the Syrian–Turkish border B2 recalled the preparation of butter. None provided information about the possibility of bringing animal products along the route except for B3. In Greece route, people bring products with them on the trip because it takes 15–25 days to reach the place where they want. Different types of cheese, canned sardines, sausages made from chicken and milk’. All these products were bought in Greek markets. No one recalled having meet veterinarians or animal health professionals in camps.

## 4. Discussion

Animals can play significant roles as economic resources, sources of food and emotional support in migration contexts [52–54], yet their function remains poorly explored. This study combined epidemiological analysis and qualitative data to assess animal presence, conceptualisation and management in camps of southeastern Europe, along the Balkan route, and the potential link with the spread of PPR and SGPX. To explore, the hypothesis that disease spread through the Balkans can be linked to human migrations we performed a disease events analysis and assessed potential spatial overlaps between outbreaks and migratory corridors. Despite their exploratory nature, the obtained maps allow to visualise the results of the analysis showing partial overlaps between migration routes and outbreak locations (Figure 2). The tracks in which diseases had spread, during the considered time frame, seem to approximately align with the routes of migrations and the locations of camps. Notably, certain regions of Bulgaria and Greece were affected by outbreaks of one of the two diseases under investigation, whereas other parts of Greece reported the co‐occurrence of PPR and SGPX. It is noteworthy that, despite an increase in the frequency and spatial spread of the diseases under consideration, human migration flows in the same region have decreased in time after the peak of more than 750.000 migrants and refugees recorded in 2015 [26, 27].

The presence of animals in refugee camps has been reported by scientific literature [52, 55–57]. Our findings confirm that small ruminants and pets are present in formal and informal settlements throughout the Balkan route, reflecting their importance for migrants. However, our qualitative analysis was unable to confirm migrants and refugees travelling the route with small ruminants or animal by‐product, as suggested by several authors [7, 8]. Instead, respondents described occasional local acquisition of livestock and ritual slaughtering during religious celebrations. For Muslim travellers, in particular, ritual animal slaughter during Islamic feasts carries deep religious and cultural significance. This practice not only marks key moments of spiritual observance but also helps preserve cultural identity and maintain a sense of continuity with their homelands throughout the journey [58–60]. The Islamic influence in the region facilitates these practices, highlighting the interaction between migrants’ traditions and the local context. Both experts and migrants reported the ritual slaughter of small ruminants. While concerns about food safety in informal camps were raised, none of the respondents identified the risks linked to ritual slaughters due to the absence of animal and meat inspections by veterinarians. The discrepancy in risk perception, is particularly relevant given the presence in the region of several endemic/emerging and re‐emerging zoonotic diseases, including Crimean‐Congo haemorrhagic fever (CCHF). This disease causes severe haemorrhagic disease in humans, while remaining asymptomatic in domestic and wild animals. Besides tick‐borne spread, direct contact with animals’ infected blood or tissues is recognised as a significant transmission pathway. Informal slaughtering practices, combined with other well‐documented risk factors, such as handling raw or undercooked meat and living in areas with high tick density, can substantially increase the likelihood of human exposure to CCHFV [61, 62].

Some authors reported a pivotal role of small ruminants in ensuring the food security of migrants by providing immediate access to milk, meat and other derivatives [52]. Our findings failed to highlight this aspect, as migrants travelling along the Balkan route seem to largely rely on food provided by NGOs or self‐bought in local markets. This evidence may be attributable to the transient nature of this migratory pathway [2]. Animals in the context of migrations play a pivotal role in the psychological support of refugees as documented by several authors [63–65]. According to some interviewed experts, human–animal interactions—particularly involving dogs and cats—represent a significant aspect of life in Balkan refugee camps. However, it was not possible to gather direct testimonies from migrants regarding their perceptions of these relationships.

The Balkan Peninsula’s and South‐Eastern Europe livestock populations are vulnerable due to their proximity to PPR–endemic areas in the Middle East and Asia [66–69], from which informal animal movements could occur. For example, smuggling of unvaccinated animals from Syria into neighbouring countries, such as Türkiye, has been reported because of war, associated embargoes and the collapse of borders and veterinary infrastructure. In the mentioned country, PPR has been officially reported in captured and slaughtered Syrian animals [7]. Effective veterinary services are critical in controlling the spread of TADs by monitoring and regulating animal movements [70, 71]. This is further supported by findings from the European project DEFEND [72], which investigated the socio‐economic drivers of lumpy skin disease and African swine fever spread into Europe, particularly in the context of political instability, poverty and porous borders. Focusing on the same study area, the project highlighted how weakened veterinary infrastructure and uncontrolled animal movements—often linked to conflict zones and migration flows—facilitate disease incursions.

The analysis performed on ‘migrants’ health policies’ revealed that the role of animals within the health frameworks governing the migrants’ settlements travelling the Balkan route is highly neglected. Current policies fail to account for the potential presence of animals in camps. As such, we can confirm that the current policies are inherently anthropocentric, as suggested by Kamenshchikova et al. [73] and in the Vétérinaires Sans Frontières (VSF) Italy policy brief [74]. This perspective reflects an underlying assumption that animals are not integral to migrant health contexts and do not directly affect human health or require interventions. Consequently, only human health professionals are involved in humanitarian support, as endorsed by national governments and UNHCR recommendations [37, 39, 40]. This exclusionary approach suggests that health threats, including zoonotic risks associated with animal presence, can be managed without applying targeted animal health measures, thus, overlooking the interconnectedness of human, animal and environmental health [75].

Such policy perspectives were perpetuated by some interviewed professionals, who worked in both formal and informal camps. The urgent challenges faced by migrants often lead volunteers and professionals to focus only on human‐centred interventions, relegating animal health welfare and other non‐human needs to a lower level of priority. However, respondents acknowledged the presence of unmanaged animals as a possible health risk (e.g., stray animals), but this was not explicitly linked to zoonotic risks. Instead, animals were more commonly perceived as a source of dirt and hygiene issues.

The anthropocentric framing of health within camps may inadvertently heighten health risks to humans by excluding animals from management frameworks [76–78]. Without adequate knowledge and management of this aspect, efforts to contain the spread of zoonotic pathogens are vain. This is especially true in the context of stray animals, which also frequently interact with both the camp environment and sylvan environment [79, 80]. Designing new health management strategies acknowledging the interconnectedness of human and animal health, thus, applying an integrated One Health approach, would promote a comprehensive understanding of health risks and create a more robust public health response within camps [75, 81, 82].

Despite historical examples of interdisciplinary approaches to migration health, such as the 1999 Missione Arcobaleno in Albania [83] and VSF Projects in Sahrawi refugee camps [84], veterinarians remain almost absent from humanitarian responses along the Balkan route. Yet their involvement is crucial for implementing holistic health policies [85].

Our findings describe healthcare along the route as fragmented, largely NGO–driven, and poorly integrated with national systems. Lack of coordinated policies perpetuates fragmented and inconsistent interventions, further undermining effective disease prevention [86]. Some policies may inadvertently contribute to gaps in responsibility, reflecting a broader hesitation to fully address the complex realities of informal migration. This limited engagement can lead to fragmented accountability for the living conditions and health challenges experienced by migrant populations [87]. As a result, there is a risk that health responses become inconsistent and uneven, potentially politicising health management and deepening existing disparities. Addressing the needs of both humans and animals in the context of formal and informal migrations would mitigate health disparities, supporting a more equitable health framework to respond to the complex social ecosystem within migration contexts. The European context presents distinct socio‐political barriers and different approaches toward migrants and refugees, reflected in policies that fail to account for each specific context, underscoring the inherent flaws of top–down approaches, which often overlook ground realities and the nuanced dynamics of the local environment [88, 89].

As mentioned by one respondent, the livestock emergency guidelines and standards (LEGS) framework may offer valuable guidance for managing animals in emergency contexts, enhancing the importance of context‐specific actions [90]. However, its recommendations are non‐binding and rarely applied in Europe. Case studies are mainly from African and Asian contexts, where traditional knowledge often recognises human–animal–environment interconnections [91–93]. In Europe, anthropocentric and Eurocentric perspectives [94] may further hinder the adoption of integrated approaches to health in migration settings indirectly promoting the spread of TADs with serious consequences which go beyond the context of migration as severe impact may be suffered by the local agro‐pastoral communities.

## 5. Conclusion

In conclusion, our findings seem to suggest that human migration along the Balkan routes is not directly responsible for the spread of the three selected TADs, as there is no evidence of significant small ruminant movements accompanying migrants. Furthermore, the opposite trends observed between migration flows and disease events, suggests that human mobility itself is unlikely the primary driver of TAD spread.

Based on these findings, we advance the hypothesis that part of the observed TADs patterns may be linked to informal small‐ruminant trade networks that exploit the same permeable border crossings used by other irregular flows. The literature consistently documents a relationship between human trafficking and animal trafficking, including shared routes, actors and logistical infrastructures [95], along the Western Balkan countries [96]. Although this evidence refers primarily to wildlife, the possibility of illegal livestock movements has also been raised in the region, as during the 2018 PPR outbreak in Bulgaria, when authorities identified unregistered small‐ruminant trade as the most likely source of virus introduction [97]. While our study cannot directly confirm this mechanism, the convergence of these elements makes the hypothesis plausible and demands further targeted investigation that should combine scientific research (e.g., to provide pathogens genomic and characterisation) embracing a transdisciplinary approach to engage local communities (e.g., for market surveys) and collaborations with local veterinary authorities (for food chain traceability).

In this context, a potential causal relationship between informal trade routes and the presence of unmanaged animals in migrants’ camps warrants investigation. In fact, the dynamicity and the complexity embedded in the Balkan corridor can add complexity on its monitoring. The current lack of cohesive policy frameworks that consider animals’ multi‐faceted roles in migrant camps marginalises both the cultural and psychological value of animals to migrants and possible risks to health. Adopting a One Health approach to bridges the policy‐practice divide, could enable a more holistic and effective response, addressing both zoonotic and TAD risks while supporting migrants’ cultural and psychological needs. This approach would necessitate collaboration among NGOs, local veterinary services, and health authorities, to foster integrated strategies that align with the realities of migration contexts. Importantly, such efforts must account for the complex interplay of migration politics, geopolitical ties and socio‐economic factors that influence implementation.

To operationalise these principles in a way that remains realistic for the Western Balkan corridor, we synthesised the findings of this study into a set of feasible One Health recommendations (Supporting Information 6). Starting from system‐level prerequisites and cross‐sectoral engagement needs, the core actions translate in a permanent One Health Cell, responsible of coordinating environmental, animal‐health, food‐safety and basic human‐health support across both formal and informal sites. This structured but flexible framework, illustrate how One Health could be practically advanced in the context even within the governance constraints that characterise migration management in the region.

By embracing an inclusive transdisciplinary One Health framework, policymakers can mitigate risks, protect livelihoods and promote the well‐being of both migrants and local communities.

Future research should prioritise the importance of collecting evidence from the voices of migrants by developing culturally sensitive and ethically sound methodologies to capture their experiences and perceptions of animals in camps while feeling safe. Participatory approaches involving migrants in designing and implementing interventions could ensure the relevance and effectiveness of proposed solutions. At the same time, involving them in such activities will enable them to introject the founding principles on which health and food safety is based in the European context.

Additionally, in‐depth studies performed by animal health professionals will be essential to understand the proximal and distal determinants of health in these contexts. Of particular importance will be to investigate the informal trading networks, existing with migrant settlements, to assess their implications for transboundary diseases spread dynamics.

Comparative economic analyses could also provide valuable insights on the costs of current migration policies versus the potential costs of a holistic management approach, providing evidence on the economic impact of transboundary diseases on local communities.

## Author Contributions

Conceptualisation: Alessandra Scagliarini, Giorgia Angeloni and Eleonora Uber. Methodology: Alessandra Scagliarini, Eleonora Uber, Alessandra Mistral De Pascali and Ludovica Ingletto. Data collection, analysis: Eleonora Uber and Ludovica Ingletto. Writing – original draft: Eleonora Uber and Alessandra Scagliarini. Writing – review and editing: Angelo Peli, Giorgia Angeloni, Ludovica Ingletto and Alessandra Mistral De Pascali.

## Funding

This study presents the results of a veterinary student Master’s thesis project and was conducted using only internal institutional resources. No external funding was received from public, commercial or not‐for‐profit agencies. Open access publishing facilitated by Universita degli Studi di Bologna, as part of the Wiley ‐ CRUI‐CARE agreement.

## Conflicts of Interest

The authors declare no conflicts of interest.

## Supporting Information

Additional supporting information can be found online in the Supporting Information section.

## Supporting information


**Supporting Information 1** Semi‐structured interview guide used to conduct qualitative interviews with field operators and experts. The guide provided a flexible script, that guided the interviews through the main thematic areas to be explored.

**Supporting Information 2** Code book developed through qualitative analysis of the interview data. It details the deductive and inductive codes used to structure the analysis and supports transparency and reproducibility of the qualitative analytical framework applied in this study.

**Supporting Information 3** Multi‐lingual questionnaire administered to migrants who explicitly stated their willingness to participate, including informed consent.

**Supporting Information 4** Biennial (non‐cumulative) outbreak maps of peste des petits ruminants (PPR) and sheep and goat pox (SGPX) covering the Period 2014–2025. Circles represent outbreak counts aggregated at the subregional level and are sized proportionally to the number of outbreaks reported within each biennium. Data retrieved from the World Animal Health Information System (WAHIS; https://wahis.woah.org/#/event-management; last accessed 01/11/2025). Outbreak counts for 2025 are updated up to 31/07/2025.

**Supporting Information 5** Annual outbreak counts of peste des petits ruminants (PPR) and sheep and goat pox (SGPX) per country, based on data retrieved from the World Animal Health Information System (WAHIS; https://wahis.woah.org/#/event-management; last accessed 01/11/2025). Outbreak counts for 2025 are updated up to 31/07/2025.

**Supporting Information 6** Summary of a three‐step One Health road map for migration contexts. Table S6 outlines foundational prerequisites, key actors to be engaged and feasible actions to be implemented through, illustrating how One Health principles can be operationalised in both formal and informal migrant settings along the Balkan route.

## Data Availability

Epidemiological data of PPR and SGPX analysed in this study are publicly available from WAHIS (https://wahis.woah.org/#/event-management) last accessed on 01/11/2025. The geospatial datasets generated and analysed during the current study, including GIS layers of outbreak distributions and migration routes, are available in the Figshare repository at: https://doi.org/10.6084/m9.figshare.31078528. Interview materials contain sensitive information and are not publicly available due to privacy constraints; de‐identified excerpts may be shared upon reasonable request and subject to ethics approval.
