# Supplementary material for: Transboundary Animal Diseases and Human Migration: A One Health Perspective on the Balkan Route
Source: Transbound Emerg Dis. 2026 Feb 13;2026:5272522. doi: 10.1155/tbed/5272522 (PMC12904845; doi:10.1155/tbed/5272522)
Supplement: Supplementary file 1 — Supporting Information 1 Semi‐structured interview guide used to conduct qualitative interviews with field operators and experts. The guide provided a flexible script, that guided the interviews through the main thematic areas to be explored. [file TBED-2026-5272522-s001.docx]

SUPPLEMENTARY MATERIAL 1- INTERVIEW GUIDE FOR OPERATOR AND EXPERTS

*The interview guide provided as Supplementary Material was developed for use with non-specialist participants. Consequently, terminology was intentionally simplified to ensure clarity and accessibility, and may not always reflect strict scientific nomenclature. This choice was made to facilitate effective communication with respondents and does not affect the scientific accuracy of the analyses or interpretations presented in the manuscript.*

Thank you for agreeing to participate in this interview, it will not take you more than 30 minutes, do you consent to the recording of the interview, which will be destroyed as soon as the project is completed? The main objective is to gather information regarding the presence of animals in migrant and refugee camps, focusing on the species present and any health issues for people, animals, food, and cross-border risk. Your responses are critical to better understand the situation and identify any necessary interventions. Your answers will be treated confidentially and anonymously; you have the right to not answer questions you find uncomfortable and at any time to withdraw from the project.

**PRELIMINARY INFORMATION**

1. In which refugee camp do you work? What is your role? What experience do you have in the migrant and refugee sector? What types of camps have you seen?
2. How long have you been working or operating in migrant and refugee camps?
3. How many facilities or camps have you visited or been assigned to in the past 10 years? Where have you worked?

**LEGISLATIVE FRAMING**

1. How do people in the camp fit into the local legislation of the country? What rights do they have? Do they have access to public health care?
2. Do you know which is it is iter which person can/will follow to be recognized refugee? If the answer is no, what are other examples? what result do they bring? Is this the situation you most frequently find yourselves dealing with?
3. What is the hierarchical organization of the camp? What are the different roles present in the camp?
4. What qualification do the workers present in the camp in which you currently operate have?

**URBAN PLANNING OF THE CAMP**

1. Where is camp x located? (proximity to pastoralist transit sites, livestock farms, markets)
2. Distance from a population center? Can people enter or leave? If yes, with what restrictions?
3. Is there a fence enclosing it? Entry controls? Is it possible for wild animals to enter? Synanthropes (e.g., mice, birds, etc.)?

**HYGIENE FOCUS**

1. People have access to the hygienic services? How are they organized? How many are there? Are they in your opinion adequate in number for the number of people?
2. What is the management of the food? Where does it come from? Are there places present where people can cook?
3. What health figures are present in the field in which you operate? if not mention veterinarians How are the veterinary systems organized in that state? Are there veterinary controls at the borders?
4. How is wastewater managed? How is waste managed?
5. Are there insects? (mosquitoes, ticks) If yes, which ones? Are pest control works done? If yes, how often?

**PRESENCE OF ANIMALS IN THE CAMP**

1. During your observations, did you notice the presence of DOMESTIC animals in the fields? If yes, which species are most common?
2. Are there any specific rules or regulations regarding the presence of animals in the fields where you operate?
3. Do you have experience with animals being introduced into the field temporarily on occasions of religious celebrations? If yes, which ones?
4. How are the animals present in the camp housed?
5. Regarding the health of the animals in the camps: have you ever noticed respiratory symptoms (e.g. nasal discharge) or sudden deaths? Diarrhea? Skin lesions? Abortions?
6. During your observations, did you notice the presence of DOMESTIC animals in the fields? If yes, which species are most common?
7. What is the general condition of the animals present in the camps? Are they properly cared for and fed? Who takes care of them?


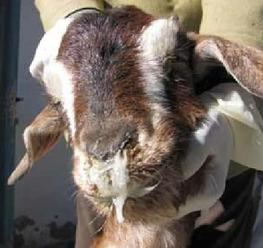

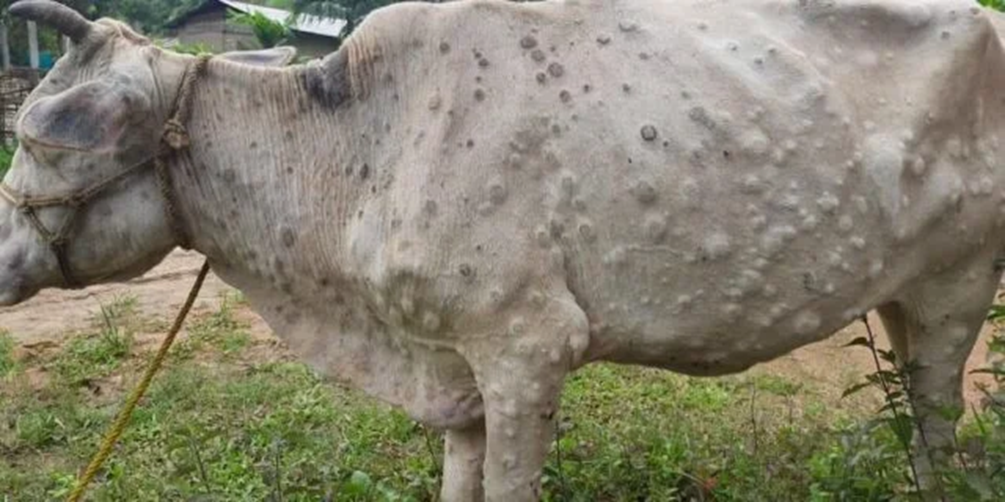

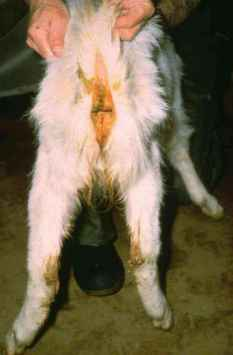


**HUMAN HEALTH**

1. Have you experienced any of the following symptoms in people in the camp? Age classes they affect? Gender?

| **Symptoms** | **Brucella** | **CCHF** | **Rift Valley Fever** |
| --- | --- | --- | --- |
| FEVER | Yes | Yes | Yes |
| HEADACHE | Yes | Yes | Yes |
| SORE MUSCLES | Yes | Yes | Yes |
| FATIGUE | Yes | Yes | Yes |
| SHIVER | Yes | Yes | Yes |
| EXCESSIVE SWEATING | Yes | Yes | Yes |
| STOMACH ACHE | Yes | Yes | Yes |
| NAUSEA | Yes | Yes | Yes |
| VOMIT | Yes | Yes | Yes |
| DIARRHEA | Yes | Yes | Yes |
| HEMORRHAGE | No | Yes | Yes |
| KIDNEY FAILURE | Possible | Possible | Possible |
| CUTANEOUS LESIONS | No | Possible | Yes |
| NEUROLOGICAL COMPLICATIONS | Possible | Possible | Possible |

1. If there was fever, what were its characteristics? Was it accompanied by respiratory symptoms (cough, cold)? Was it recurrent?
2. Did you encounter any situations of health issues related to the animals in the camps? If yes, what were the main issues encountered? Sheep and goat focus
3. Did you notice correlations between the health conditions of animals and people in the camps?

**FOOD SAFETY**

1. How are food and food resources managed in the camps? What is the origin of the food? How is the water supply managed?
2. Are artisanal cheeses produced? (e.g., Baladi, Kanafa, Jibneh Arabieh, ackawi, halloumi, labneh)
3. Are religious holidays celebrated? If yes, how?
4. Are slaughterings done in the fields? If yes, are there veterinarians?

**FURTHER CONSIDERATIONS**

1. Do you have any additional observations or considerations regarding the presence of animals in migrant and refugee camps that you would like to share?
2. Do you have specific recommendations to improve the management of the presence of animals in camps and reduce risks to human and animal health?

Thank you again for your participation and valuable information. Your answers will contribute significantly to the understanding and management of issues related to the presence of animals in migrant and refugee camps.
