# Supplementary material for: Transboundary Animal Diseases and Human Migration: A One Health Perspective on the Balkan Route
Source: Transbound Emerg Dis. 2026 Feb 13;2026:5272522. doi: 10.1155/tbed/5272522 (PMC12904845; doi:10.1155/tbed/5272522)
Supplement: Supplementary file 2 — Supporting Information 2 Code book developed through qualitative analysis of the interview data. It details the deductive and inductive codes used to structure the analysis and supports transparency and reproducibility of the qualitative analytical framework applied in this study. [file TBED-2026-5272522-s002.docx]

# Supplementary Material 2: Codebook and main themes

This codebook is organised into two sections: Table S2A Deductive codes, derived a priori from the Socio-Ecological System Framework (SESF), and Table S2B Inductive codes, emerging from interview data. Each code includes its definition, representative quote, and corresponding SESF level(s).

TableS2C presents the main themes identified through this coding process.

## Table S2A. Deductive Codebook (SESF-based codes)

| Code Name | SESF Variable / Level | Definition / Description | Example Quote |
| --- | --- | --- | --- |
| Knowledge of Hygiene and Health Practices | Water resources; Camp and Camp-like settings / Individual; Organisational; interpersonal; | Awareness and practices related to hygiene, health and disease prevention. | “People knew to boil water, but often there was no soap or access to showers.” |
| Poor Hygiene and sanitary conditions | Camp and Camp-like settings; Housing standards; Presence of vectors / Individual; Organisational; interpersonal; | Overcrowding, absence of waste management practice, absence of running water (informal camps), heavy presence of pests |  |
| Animals in Squats and Camps | Animal presence / Individual; Community | Presence of animals in formal and informal settlements. | “Stray dogs are adopted by the people residing in the camps” |
| Veterinary and Animal Management Gap | Animal presence; Politics/ Organisational; Policy | Absence of any veterinary or animal-health component in humanitarian action. |  |
| Neglect of Animal Health in Policy | Policy | Lack of institutional recognition of animal presence or welfare in migration frameworks. | “There are no guidelines for animals in the camps.” |

## TableS2B. Inductive Codebook (Emergent Codes, SESF-linked)

| Code Name | Linked SESF Level(s) | Definition / Description | Example Quote |
| --- | --- | --- | --- |
| Personal Relation with Animals | Individual; Interpersonal | Emotional bonds or practical uses of animals during displacement (companionship, food source). | “They took care of a mother pitbull and her puppies; it was an emotional investment.” |
| Coping Mechanisms | Individual;  Community | Strategies to cope with stress and uncertainty, often through caring for animals or social ties. | “Feeding cats made the camp feel more human; it gave people something to do every day.” |
| Solidarity Networks | Interpersonal;  Community | Informal help among migrants and between migrants and locals (sharing food, clothes, or shelter). | “Neighbours allowed them to charge phones and often brought food.” |
| Cultural Continuity | Community;  Organisational | Maintenance of traditions (religious meals, animal slaughter, communal cooking). | “At the end of Ramadan, friends bought a sheep; they kept traditions alive even there.” |
| Food Procurement | Individual;  Community;  Organisational; | Food distribution and/or food procuremente in informal and formal camps | “We had a warehouse; donations arrived by truck, and we bought fresh food locally.” |
| Humanitarian Health Services | Politics/Organisational | NGO or volunteer initiatives for basic medical care (“street medicine”, MSF, Red Cross). | “MSF enters twice a week; before, the Red Cross was there every day. In informal camps there was only us (NGO)” |
| Informal Camp Governance | Community;  Organisational:  Interpersonal | Management of squat rules, sanitation, or leadership roles among residents. | “Some squats had a stable group acting as reference for newcomers.” |
| Access to Health Systems | Policy;  Organisational | Barriers for migrants to access healthcare and discrimination. | “We knew hospitals wouldn’t take them seriously, even when we accompanied them” |
| Border Management and Repression | Policy;  Community | Institutional practices (pushbacks, eviction, sealing of squats). | “Police destroyed the squats and sealed them; people kept moving constantly.” |
| Humanitarian Space Restrictions | Policy;  Organisational | Governmental control over NGO access to camps and visibility of conditions. | “Only registered organizations can enter; they don’t want too many eyes inside.” |
| Perceived Health Risks | Individual | Awareness of physical and mental health issues faced by migrants (e.g., scabies, injuries, exhaustion). | “We often treated skin infections and scabies; people slept on the floor without heating.” |

## TableS2C. Main themes emerged from the interviews

| Theme | Interpretive Description | Main SESF Level(s) |
| --- | --- | --- |
| 1. Human–animal relationship for emotional welfare | Animals provided companionship, routine, and emotional relief in unstable environments. Their presence helped migrants reconstruct a sense of normality and control amid uncertainty, functioning as informal psychosocial support. | *Individual;*  *Interpersonal;*  *Community* |
| 2. Cultural continuity through ritual and food | The slaughter of small ruminants during religious festivities (Eid al-Adha) and the exchange of animal-derived foods (meat, cheese) sustained collective identity and spiritual practices. | *Community;*  *Organisational;*  *Policy* |
| 3. Institutional invisibility of animals | Despite their ubiquity, animals remain absent from humanitarian and health governance. The deliberate omission of veterinary oversight was described as “wilful neglect,” reflecting institutional denial and avoidance of responsibility. | *Organisational;*  *Policy* |
| 4. Health hierarchies and competing priorities | Humanitarian actors and migrants prioritised acute human needs (healthcare, food, hygiene) over animal or environmental health. Fragmented NGO-based care, discrimination in hospitals, and systemic under-resourcing revealed unequal health hierarchies and eroded trust in institutions. | *Interpersonal;*  *Organisational;*  *Policy* |
| 5. Fragile ecosystems of displacement | Overcrowding, poor waste management, and limited sanitation created ecologically unstable environments where humans, animals, and pests coexisted. | *Community;*  *Organisational;*  *Policy* |
