# Supplementary material for: Transboundary Animal Diseases and Human Migration: A One Health Perspective on the Balkan Route
Source: Transbound Emerg Dis. 2026 Feb 13;2026:5272522. doi: 10.1155/tbed/5272522 (PMC12904845; doi:10.1155/tbed/5272522)
Supplement: Supplementary file 3 — Supporting Information 3 Multi‐lingual questionnaire administered to migrants who explicitly stated their willingness to participate, including informed consent. [file TBED-2026-5272522-s003.pdf]

Information on Animals and Migrations

Hello,I am inviting you to participate in this questionnaire as part of my research on the role of animals for people on the move along the Balkan route. I would like to gather your experience, and it will take only 6 minutes to complete. The data will be collected and processed completely anonymously. By filling out this form, you confirm your willingness to participate in this research . You can withdraw at any time by closing the form. For any questions or further information, feel free to contact me:Eleonora
Phone: +39 380 865 6524
Email: eleonora.uber@studio.unibo.itThank you!

1

Which country are you from?

2

In which year did you leave your country of origin?

3

Which countries have you traveled through?

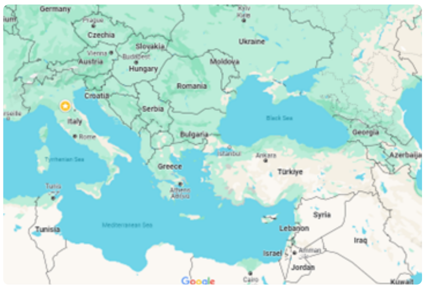

4

During your journey, did you meet anyone who had brought animals with them? If so, which animals and how many? Could you specify their origin? (e.g., purchased locally, bought in another country, etc.)

5

Where did you encounter them? If it happened more than once, please specify.

6

During your journey, did you meet anyone who brought animal-based products (such as meat, milk, or dairy products like cheese, e.g., Labneh)? If yes, where? Which products and how much? Were they homemade or industrial? See example in the photo

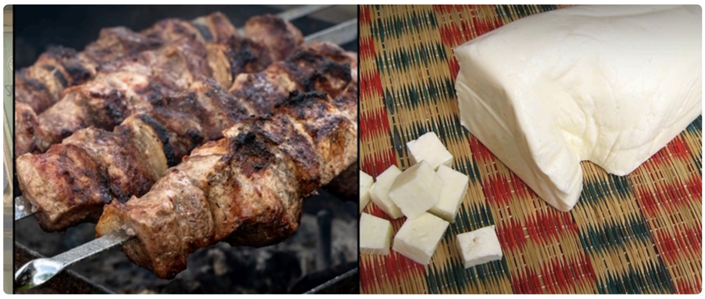

7

During your journey, did you ever celebrate religious occasions that involved the consumption of animal-based products (e.g., Eid al-Fitr)? Could you briefly explain how?

8

Have you ever seen these animals in the places where you stopped during your journey?

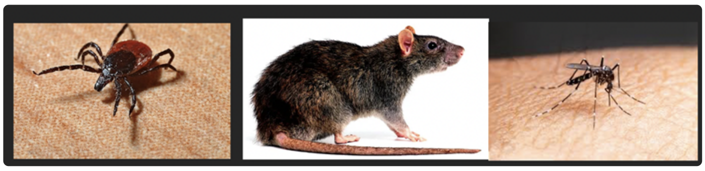

9

Have you ever seen these symptoms in the animals you encountered during your journey? (nasal discharge, skin lesions, diarrhea, abortion)

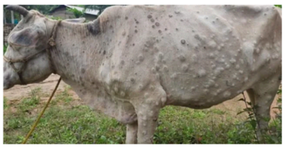

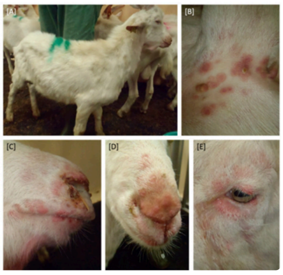

10

What healthcare professionals did you encounter during your journey? (e.g., doctors, nurses) Did you meet any veterinarians?

11

Would you be interested in answering additional questions (in a group interview) about the animals you encountered during your journey?

- ○ Yes
- ○ No

12

Please leave a contact where you can be reached, or you can contact +380 8656524 or eleonora.uber@studio.unibo.it.

Questo contenuto non è stato creato né approvato da Microsoft. I dati che invii verranno recapitati al proprietario del modulo.

Microsoft Forms
