# Supplementary material for: Transboundary Animal Diseases and Human Migration: A One Health Perspective on the Balkan Route
Source: Transbound Emerg Dis. 2026 Feb 13;2026:5272522. doi: 10.1155/tbed/5272522 (PMC12904845; doi:10.1155/tbed/5272522)
Supplement: Supplementary file 4 — Supporting Information 4 Biennial (non‐cumulative) outbreak maps of peste des petits ruminants (PPR) and sheep and goat pox (SGPX) covering the Period 2014–2025. Circles represent outbreak counts aggregated at the subregional level and are sized proportionally to the number of outbreaks reported within each biennium. Data retrieved from the World Animal Health Information System (WAHIS; https://wahis.woah.org/#/event-management; last accessed 01/11/2025). Outbreak counts for 2025 are updated up to 31/07/2025. [file TBED-2026-5272522-s004.docx]

**SUPPLEMENTARY MATERIAL 4** – Biennial (non-cumulative) outbreak maps of PPR and SGPX (2014–2025)

Circles represent outbreak counts aggregated at subregional level and are sized proportionally to the number of outbreaks reported during each biennium. Data retrieved from WAHIS (<https://wahis.woah.org/#/event-management>. Last accessed 1/11/2025). The 2025 outbreak count is updated until the 31/07/2025.


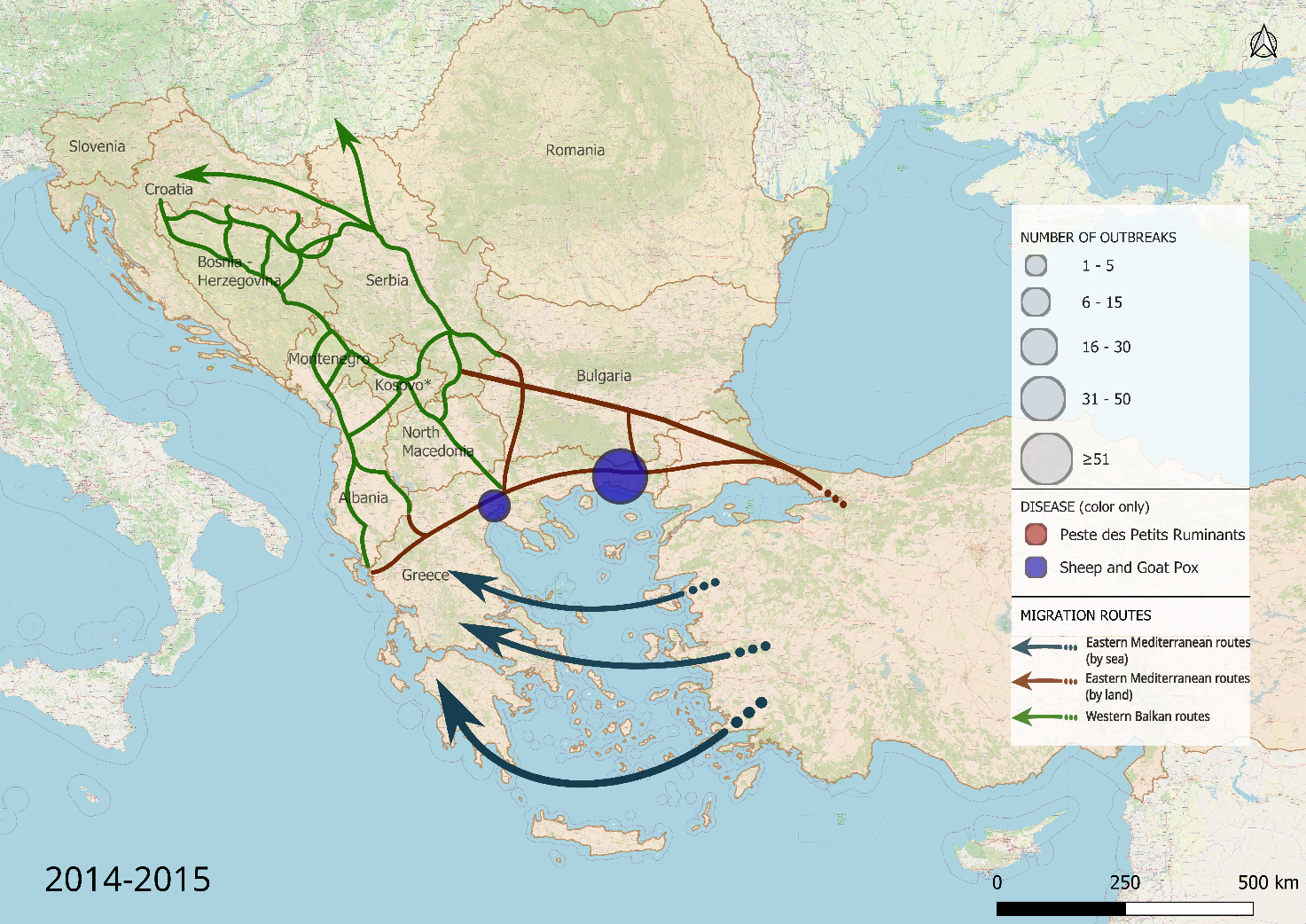


*Figure S4A. Outbreaks of PPR and SGPX, 2014–2015.*


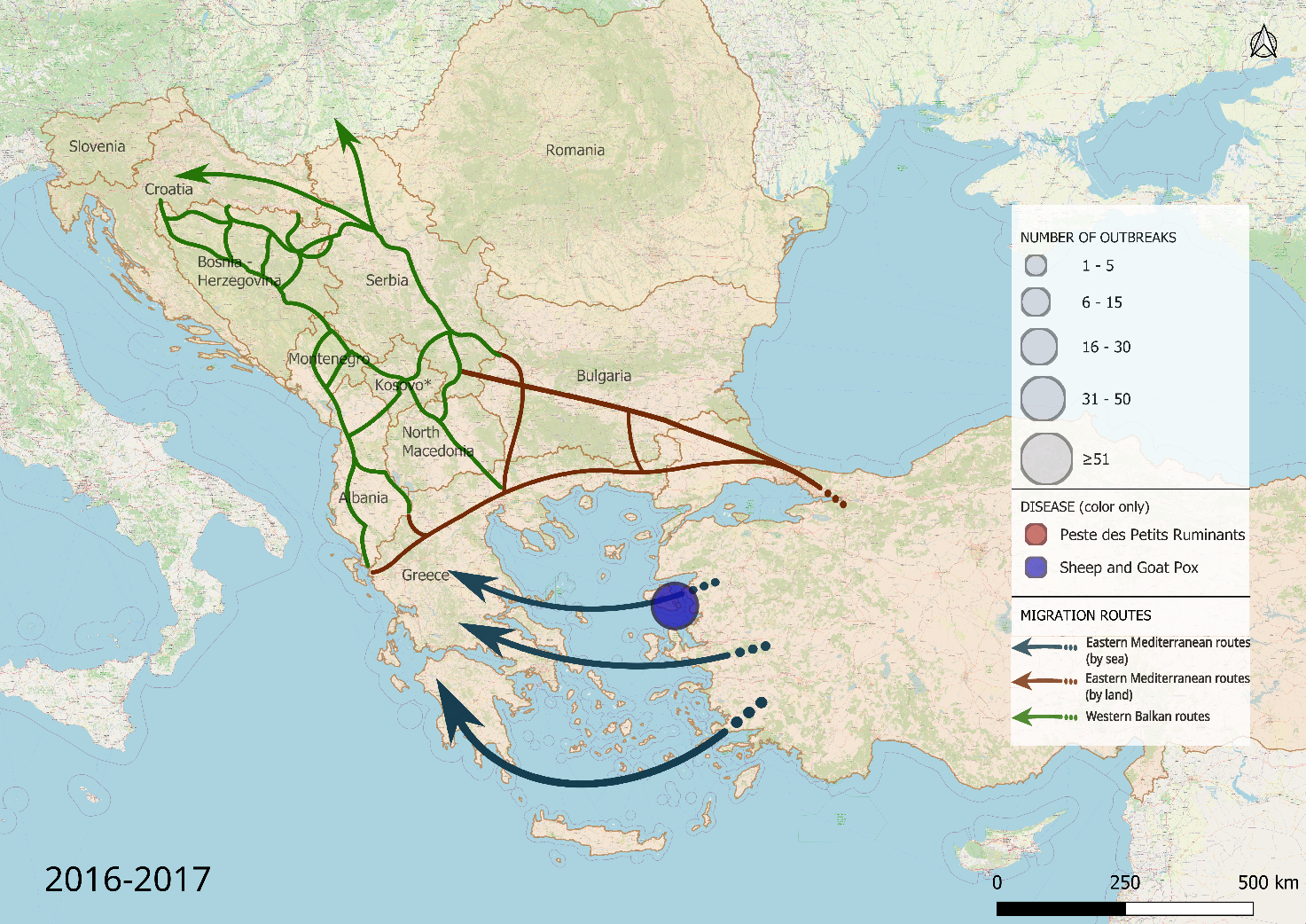


*Figure S4B. Outbreaks of PPR and SGPX, 2016–2017.*


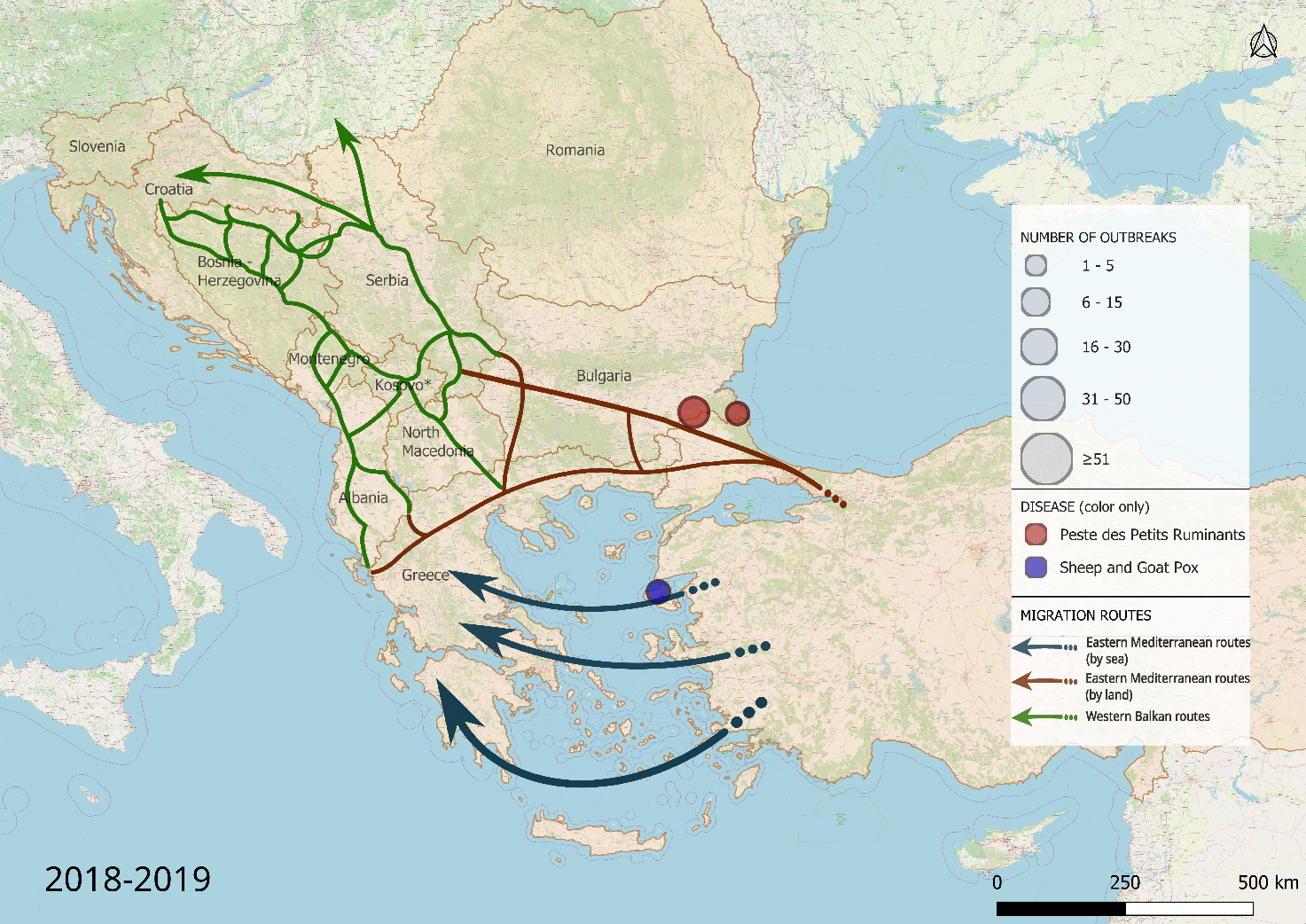


*Figure S4C. Outbreaks of PPR and SGPX, 2018–2019.*


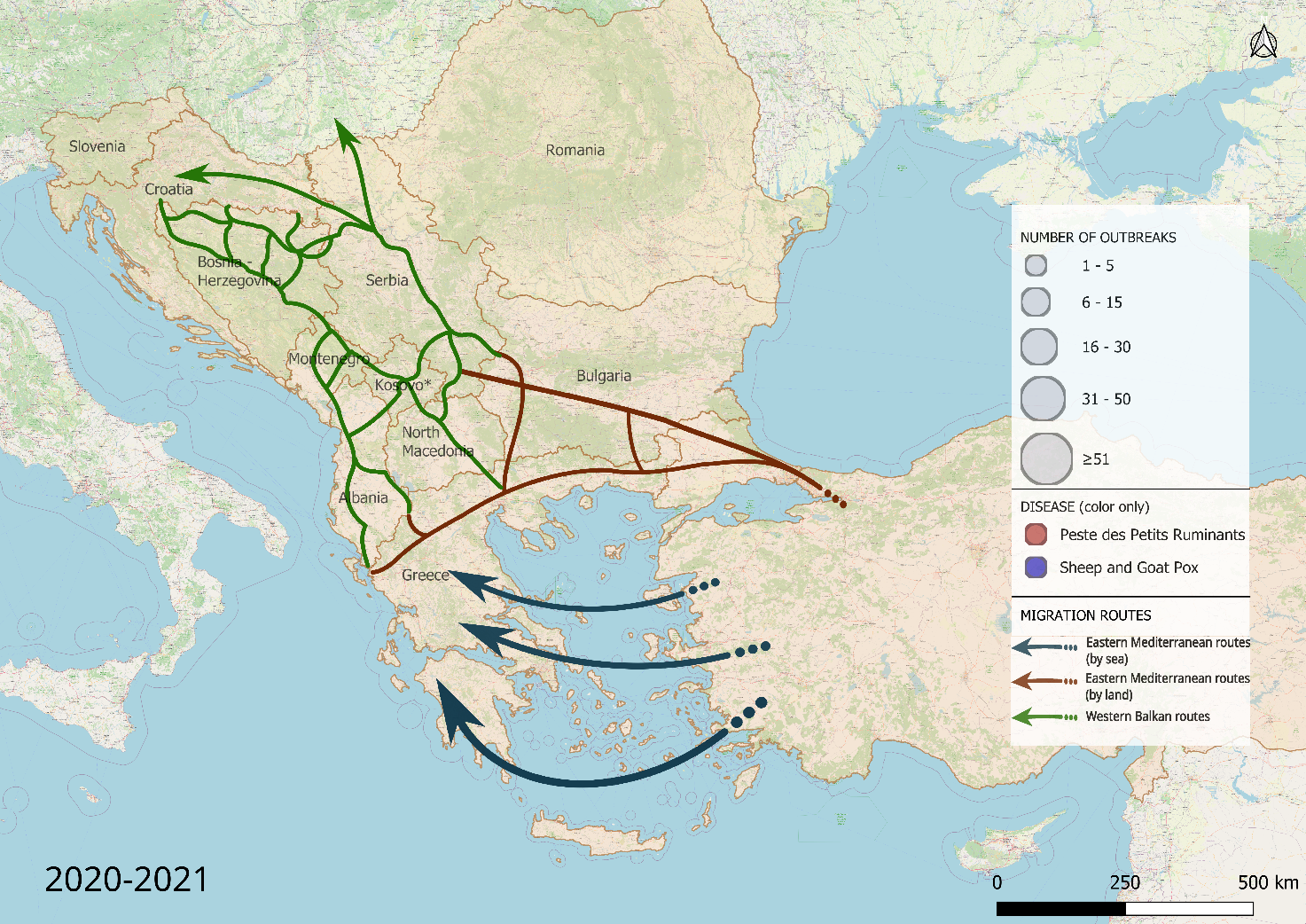


*Figure S4D. Outbreaks of PPR and SGPX, 2020–2021.*


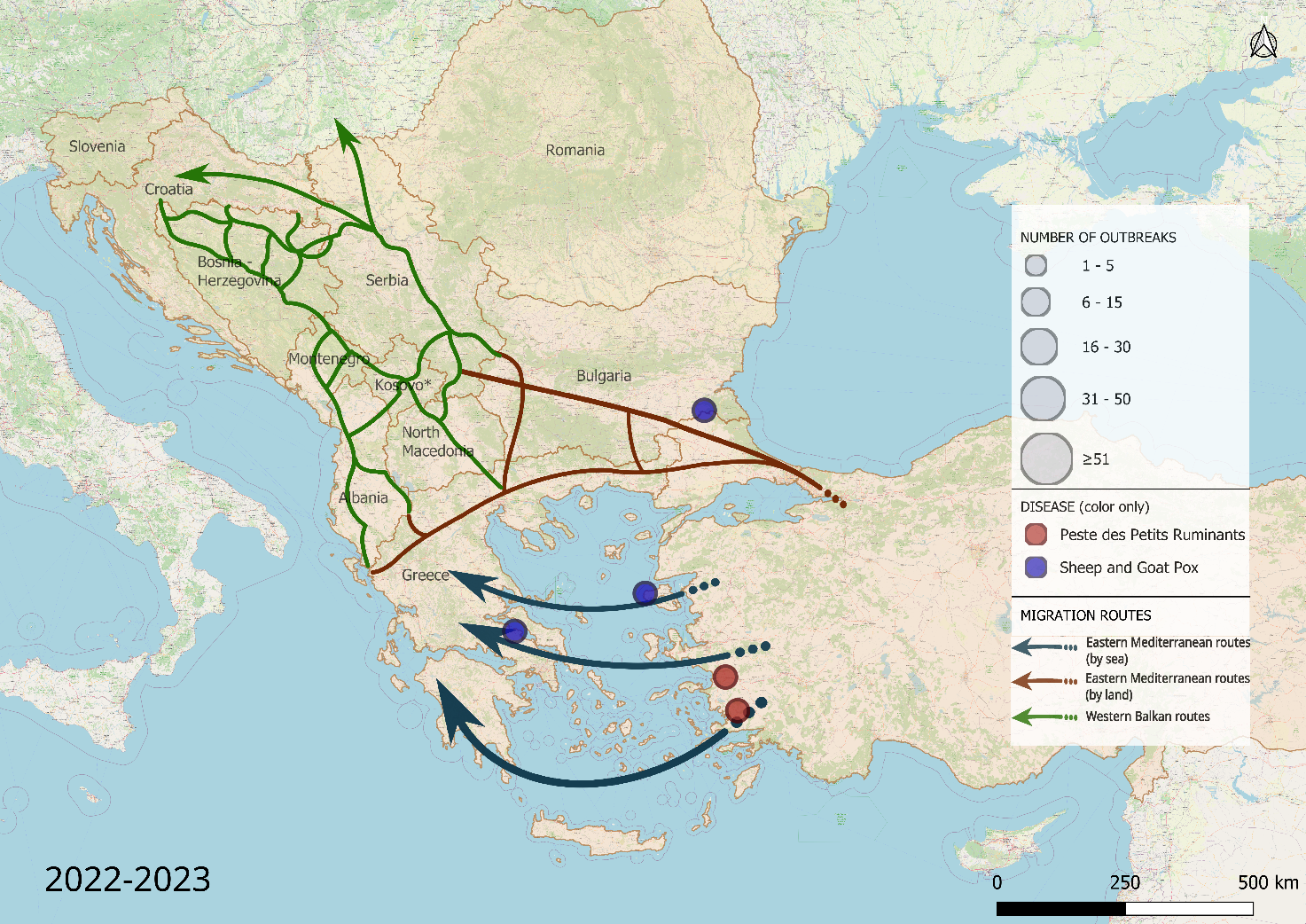


*Figure S4E. Outbreaks of PPR and SGPX, 2022–2023.*


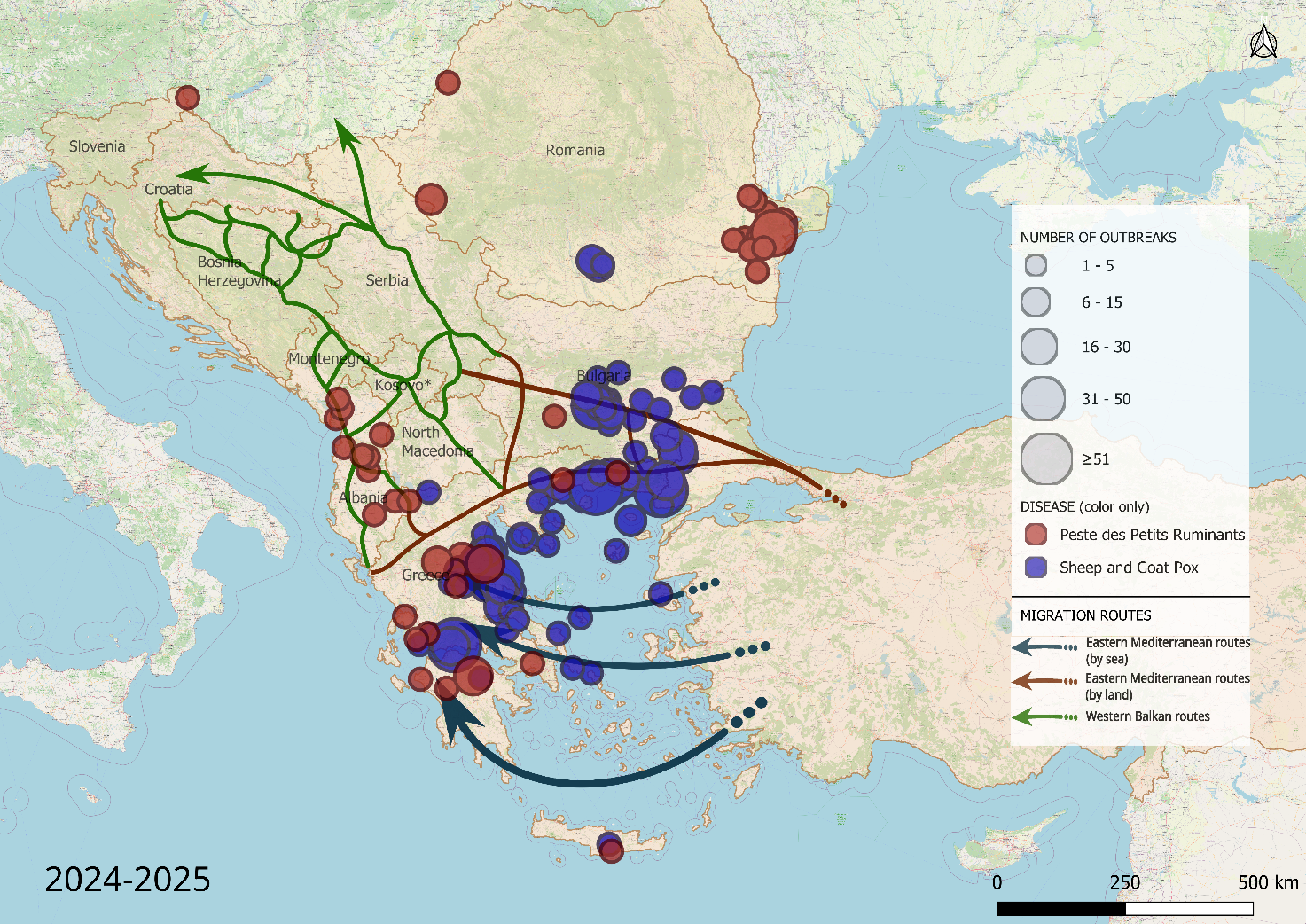


*Figure S4F. Outbreaks of PPR and SGPX, 2024–2025.*
