# Supplementary material for: Transboundary Animal Diseases and Human Migration: A One Health Perspective on the Balkan Route
Source: Transbound Emerg Dis. 2026 Feb 13;2026:5272522. doi: 10.1155/tbed/5272522 (PMC12904845; doi:10.1155/tbed/5272522)
Supplement: Supplementary file 5 — Supporting Information 5 Annual outbreak counts of peste des petits ruminants (PPR) and sheep and goat pox (SGPX) per country, based on data retrieved from the World Animal Health Information System (WAHIS; https://wahis.woah.org/#/event-management; last accessed 01/11/2025). Outbreak counts for 2025 are updated up to 31/07/2025. [file TBED-2026-5272522-s005.docx]

Supplementary Material 5 -Annual outbreak count per Country

**Table S5A**: number of PPR outbreaks per year in the considered countries, according to data available on WAHIS (<https://wahis.woah.org/#/event-management>. Last accessed 1/11/2025). The 2025 outbreak count is updated until the 31/07/2025.

| Country | 2014 | 2015 | 2016 | 2017 | 2018 | 2019 | 2020 | 2021 | 2022 | 2023 | 2024 | 2025 |
| --- | --- | --- | --- | --- | --- | --- | --- | --- | --- | --- | --- | --- |
| Albania | 0 | 0 | 0 | 0 | 0 | 0 | 0 | 0 | 0 | 0 | 0 | 13 |
| Bulgaria | 0 | 0 | 0 | 0 | 7 | 0 | 0 | 0 | 0 | 0 | 1 | 0 |
| Greece | 0 | 0 | 0 | 0 | 0 | 0 | 0 | 0 | 0 | 0 | 86 | 0 |
| Hungary | 0 | 0 | 0 | 0 | 0 | 0 | 0 | 0 | 0 | 0 | 0 | 3 |
| Romania | 0 | 0 | 0 | 0 | 0 | 0 | 0 | 0 | 0 | 0 | 67 | 1 |
| Türkiye (Rep. of) | 0 | 0 | 0 | 0 | 0 | 0 | 0 | 0 | 0 | 3 | 0 | 0 |

**Table S5B**: number of SGPX outbreaks per year in the considered countries, according to data available on WAHIS (<https://wahis.woah.org/#/event-management>. Last accessed 1/11/2025). The 2025 outbreak count is updated until the 31/07/2025.

| Country | 2014 | 2015 | 2016 | 2017 | 2018 | 2019 | 2020 | 2021 | 2022 | 2023 | 2024 | 2025 |
| --- | --- | --- | --- | --- | --- | --- | --- | --- | --- | --- | --- | --- |
| Bulgaria | 0 | 0 | 0 | 0 | 0 | 0 | 0 | 0 | 0 | 1 | 11 | 105 |
| Greece | 85 | 0 | 2 | 29 | 4 | 0 | 0 | 0 | 0 | 5 | 328 | 406 |
| Romania | 0 | 0 | 0 | 0 | 0 | 0 | 0 | 0 | 0 | 0 | 0 | 21 |
| Türkiye (Rep. of) | 0 | 0 | 0 | 0 | 0 | 0 | 0 | 0 | 0 | 0 | 0 | 1 |
