# Supplementary material for: Transboundary Animal Diseases and Human Migration: A One Health Perspective on the Balkan Route
Source: Transbound Emerg Dis. 2026 Feb 13;2026:5272522. doi: 10.1155/tbed/5272522 (PMC12904845; doi:10.1155/tbed/5272522)

| **Supplementary Material 6 – Actions to implement a holistic approach to migration**  Table S6A summarises a three-step One Health roadmap: the foundational prerequisites, the actors to be engaged, and the feasible actions to be implemented through a permanent One Health Coordination. This structure shows how One Health principles can be practically applied in both formal and informal migrant settings. | | |
| --- | --- | --- |
| **Rationale (One Health Lens steps)** | | **Recommended Actions** |
| **1. System-level prerequisites**  Migrant health risks arise from the interaction of social determinants, environmental hazards, animal presence, and vector ecology. These interdependencies require an intentional One Health orientation rather than fragmented, sector-specific responses. | | • Integrate One Health competencies across stakeholders (Governments, NGOs, public health services, migrants)  • Promote equitable access to health for people in both formal and informal sites.  • Recognise and de-criminalise animal ownership and ritual slaughter, supporting safe practices rather than policing them. |
| **2. Cross-sectoral engagement**  Informal sites are mainly managed by NGOs and grassroots actors; without environmental-health and animal-health expertise, risks remain unmanaged. Migrant leaders and religious associations facilitate culturally trusted communication. | | • Expand healthcare teams to include animal-health and environmental-health professionals  .• Engage migrants and community leaders as peer educators and co-producers of health.  • Collaborate with faith-based organisations to support trust and communication. |
| **3. One Health Cell (coordination mechanism)**  Fragmentation between formal and informal settings leads to inconsistent management of environmental, human, and animal health risks. A stable coordination mechanism can ensure continuity, cross-notification, and proportional support. | | • Establish a *One Health Cell* active across formal and informal sites.  • In formal sites: include public veterinary services, public health staff, and environmental health officers.  • In informal sites: rely on NGO staff integrating an animal-health consultant and environmental-health technician.  • Maintain continuous coordination between both cells to ensure communication, shared risk assessment, and aligned responses. |
| **ONE HEALTH CELL** | | |
| **Topic of intervention** | **Rationale** | **Recommended Actions** |
| **4. Environmental & animal-health management** | Environmental exposures (wastewater, refuse, cooking areas), synanthropic species, and interactions with animals influence foodborne, waterborne, and vector-borne disease dynamics. | • Implement basic environmental health measures (waste points, wastewater drainage, safer cooking setups).  • Promote risk-based synanthropic animals management (rats, mosquitoes, ticks).  • Adopt non-criminalising strategies for animals (health check, vaccination/sterilisation when feasible). |
| **5. Safe food systems & ritual slaughter** | Informal food preparation and ritual slaughter are common cultural practices; if unsupported, they increase risks rather than reducing them. | • Provide guidance and hygiene toolkits for safer slaughter, without formalising or restricting practices.  • Organise “safe slaughter windows” during religious and culturally significant periods.  • Promote basic food and water hygiene education through peer educators. |
| **6. Human health support in informal sites** | Informal sites lack institutional healthcare, creating gaps in early detection of illness, triage, and referral. Addressing this absence is essential to maintain continuity across human, animal, and environmental health domains. | • Provide basic human health triage and care. • Offer first aid and symptoms screening. • Establish referral pathways to formal services for urgent or complex cases. • Perform syndromic surveillance. • Ensure two-way communication between informal-site teams and formal health services. |

Figure S6B summarises action and actors involved in the presented holistic approach to migration, as explained in Table 6S.
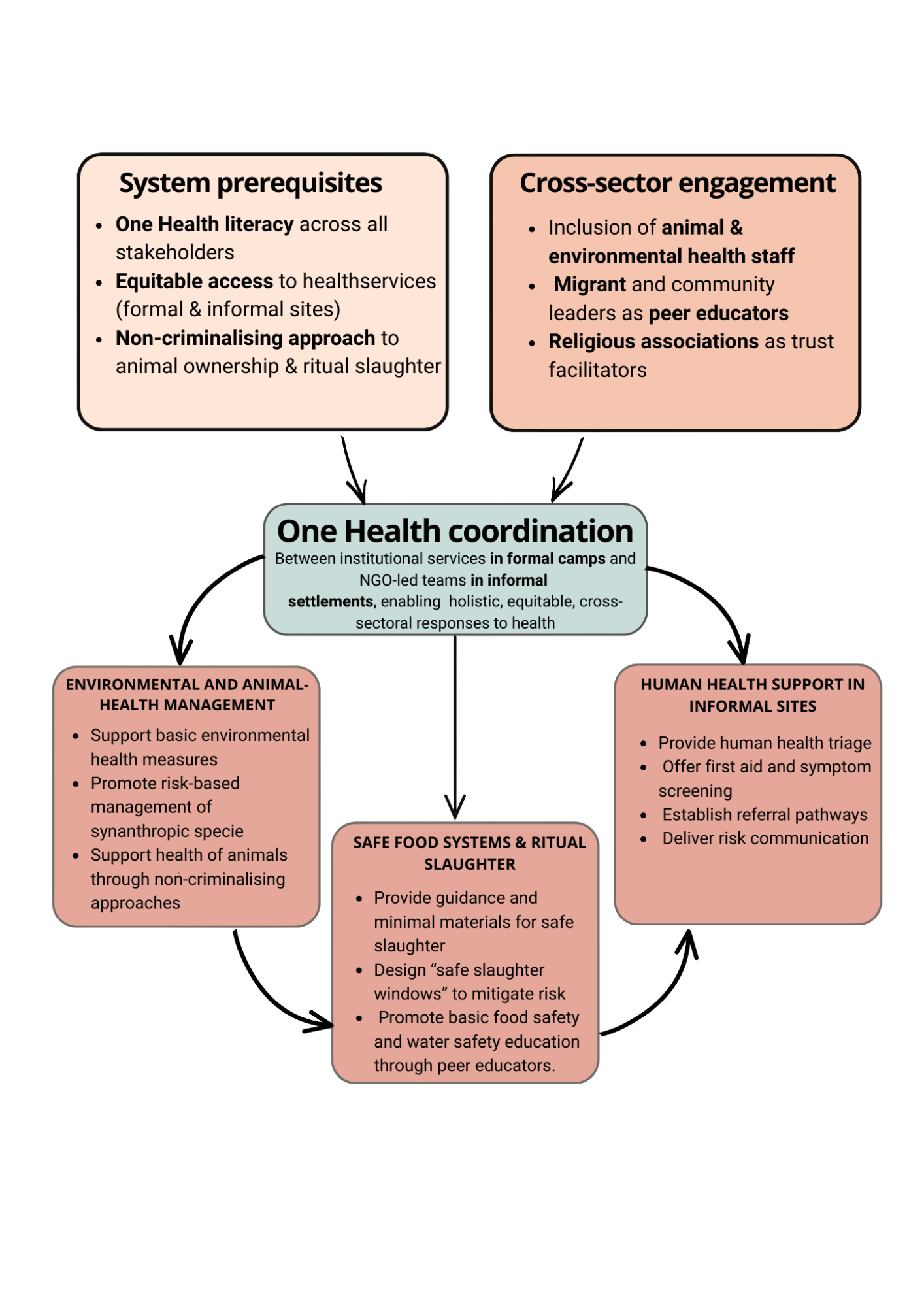

Supplement: Supplementary file 6 — Supporting Information 6 Summary of a three‐step One Health road map for migration contexts. Table S6 outlines foundational prerequisites, key actors to be engaged and feasible actions to be implemented through, illustrating how One Health principles can be operationalised in both formal and informal migrant settings along the Balkan route. [file TBED-2026-5272522-s006.docx]
